# The relationship between serum levels of surfactant protein D in COPD exacerbation severity and mortality

**DOI:** 10.3906/sag-1809-6

**Published:** 2019-06-18

**Authors:** Fatma Esra GÜNAYDIN, Nurdan KALKAN, Gülşah GÜNLÜOĞLU, Esma Nur AKTEPE, Barış DEMİRKOL, Sedat ALTIN

**Affiliations:** 1 Department of Allergy and Immunology, Faculty of Medicine, Uludağ University, Bursa Turkey; 2 Department of Pulmonary Diseases, Health Sciences University Yedikule Hospital for Chest Disease and Thoracic Surgery, İstanbul Turkey

**Keywords:** COPD, exacerbation, surfactant protein D

## Abstract

**Background/aim:**

Chronic obstructive pulmonary disease (COPD) is a major cause of morbidity and mortality. In COPD patients, various inflammatory markers such as cytokines and acute phase proteins, which show systemic inflammation in the circulation, increase during exacerbations. In our study, we aimed to determine the relationship between serum SP-D levels and exacerbation severity, clinical course of the disease, and early mortality after discharge.

**Materials and methods:**

Fifty hospitalized patients with COPD acute exacerbation (46 male and 4 female) were recruited in this study. Thirty-three of the subjects (31 male and 2 female) were reevaluated after discharge. Venous blood samples were taken from all patients and followed up for exacerbation frequency, hospital admission, and mortality for 12 months.

**Results:**

Serum SP-D levels in the stable period of the patients were lower than exacerbation (P < 0.001). The median exacerbation period SP-D level of the patients admitted to emergency department in the first month was statistically significantly higher than that of the patients who were not admitted (P < 0.05) after discharge. There was a correlation between the rate of emergency admission and serum SP-D levels during the 12-month period after discharge (P = 0.04 (r = 0.29)).

**Conclusion:**

Our study showed that serum SP-D was found to be a useful biomarker in predicting emergency admission and predictor of the health status of COPD patients but did not predict early mortality after the exacerbation.

## 1. Introduction

Chronic obstructive pulmonary disease (COPD) is one of the major causes of morbidity and mortality worldwide (1,2). COPD is a global health burden that affects 10% of the world’s population and results in 3 million deaths and $44 billion in health care costs annually (3). Until recently, COPD had been defined as a pulmonary disease characterized by progressive airflow limitation, whereas it has recently been observed that airflow limitation is associated with abnormal inflammatory response and is not only limited to have effects on the lungs but also has systemic effects.

COPD exacerbations are frequently associated with increased airway inflammation, mucus production, and significant air trapping. A COPD exacerbation is defined as ‘acute worsening of respiratory symptoms in a way that results in the need for additional treatment’ (1). It negatively affects the state of health, hospital admission, and course of the disease (1). Various inflammation marker levels, such as cytokines, chemokines, and acute phase proteins, which exhibit systemic inflammation in the blood, are increased in correlation with the severity of the disease, particularly during the exacerbation periods in patients with COPD (4).

Surfactant protein D (SP-D), a member of the collagen-dependent C-type lectins or collectins, is a large hydrophilic protein synthesized by collagenous glycoprotein in Type 2 pneumocytes (5). It plays a role in local alveolar defense mechanisms. Its most important advantage is that it can be detected in the serum as a lung-specific marker (2). Increase in the serum level is also observed in cases with pulmonary fibrosis, asthma, and ARDS, which progress with chronic inflammation in smokers (6).

SP-D is considered to play an important role in the pathogenesis of COPD. SP-D is a biomarker that can be used to determine a worsening of the state of health and decreasing pulmonary functions in patients with pulmonary diseases (7). In previous studies, COPD exacerbations were associated with high expressions of SP-D, and the serum SP-D levels were notably decreased 30 days after the beginning of exacerbation (8,9).

Among the studies conducted thus far, there has been no study that examines the relationship between SP-D and mortality due to COPD exacerbation during hospitalization or in the early period after discharge. In our study, we aimed to determine the relationship between the serum SP-D level as a pulmonary-specific inflammation marker and the exacerbation severity level, clinical course and disease morbidity, and mortality during the early period after discharge in patients who were hospitalized because of a COPD exacerbation.

## 2. Materials and methods

In this study, patients diagnosed with COPD and who were admitted to the emergency services because of exacerbation between January 2016 and January 2017 at the Yedikule Chest Diseases and Thoracic Surgery Training and Research Hospital were evaluated. The study group comprised 50 patients who met the inclusion criteria. The study was approved by the institutional ethics committee of Yedikule Chest Hospital Disease Hospital. Informed consent was obtained from each participant. Patients who had a COPD exacerbation were evaluated during the exacerbation and 30–45 days after the exacerbation, i.e. during the stable period.

The study group included 50 patients with COPD exacerbation who were admitted to the emergency services and they were hospitalized because of worsening of their respiratory symptoms (dyspnea, increase in the amount of sputum, and/or darkening of the sputum) to the point that they needed additional treatment. All of the patients were diagnosed with COPD according to GOLD prior to the exacerbation and were aged between 40 and 75 years. Patients who had a tumor, pneumonia, or disseminated bronchiectasis findings on pulmonary imaging, who had a known organ malignancy, who were taking immunosuppressive treatments and who did not accept being included in the study were excluded from the study. Patients who had pulmonary pathologies other than COPD (interstitial pulmonary disease, asthma, and bronchiectasis), chronic kidney failure, and chronic lung failure were also excluded from the study.

All patients followed up for exacerbation frequency, hospital admission, and mortality for 12 months after discharge. We planned to reevaluate the included patients’ serum SP-D level and other parameters during the stable period, i.e. 30–45 days after discharge. However, only 33 patients could be reevaluated during the stable period because of various reasons: 11 patients were not stable after discharge and needed rehospitalization, two patients died after discharge and four patients discontinued follow-up.

### 2.1 Follow-up and evaluations

The ‘informed consent form’ was signed by all patients included in the study or their legally responsible relatives. The case report form was filled in in the first 24 h of hospitalisation. Identity information, complaints, smoking anamnesis, vital findings, physical examination findings, lung radiography findings, pulmonary function tests, mMRC dyspnea scale, CAT score, treatment method, hospitalization time, and blood biochemistry results were recorded in the case report form. In the stable period (i.e. 30–45 days after discharge) we reevaluated pulmonary function tests, mMRC dyspnoea scale, CAT score, CBC, and blood biochemistry results.

### 2.2. Measurement of biochemical parameters

A venous blood sample was obtained from each patient. The venous peripheral blood specimen (5 mL) was added to a tube with K3-EDTA for CBC (complete blood count) analysis and to an additive-free tube for analysis of C-reactive protein (CRP) and SP-D levels. Serum samples were prepared by collecting blood in a vacuum tube and allowing it to clot for 30 min at 24 °C. Approximately 2 mL of serum was obtained after centrifugation at 3500 g for 10 min and stored in small aliquots at –80 °C until analysis. The SP-D level was studied by the ELISA immunoassay method. The serum CRP level was measured using a Beckman Coulter AU2700 plus (Olympus Corporation, Tokyo, Japan), CBC was conducted using an Abbott Cell-Dyne® 3700 System device (Abbott Diagnostics, Santa Clara, CA, USA). Blood collection was performed on the first day of hospitalization, and spirometry was performed 30–45 days after the onset of exacerbation, i.e. when the patients were in the stable period.

### 2.3. Pulmonary function test

The pulmonary function test was performed using a clinical Spirometer (Vmax Vyntus SPIRO, Hoechberg, Germany), in accordance with the ERS standards. We conducted forced vital capacity (FVC) and forced expiratory volume (FEV1) measurements and calculated the FEV1/FVC ratio.

### 2.4. Statistical analysis

The study was conducted on the data of 50 patients included in the study during the COPD exacerbation period and 33 patients out of these 50 patients were evaluated during the stable period. Data were completed by being transferred into the SPSS 23.0 (IBM Corp, Armonk, NY, USA) programme. Out of the measures of central tendency, the mean, median, and IQR standard deviation values were provided for numerical variables and frequency distributions (number and percentage) were provided for categorical variables. Nonparametric tests were preferred as the numerical variables that were not compatible with the normal distribution according to the Kolmogorov–Smirnov test. The difference between the categorical variables of the two groups was analyzed using the Mann–Whitney U test; if the number of groups was more than two, the Kruskal–Wallis test was used. Wilcoxon’s test was used for comparison of data acquired during the exacerbation and stable periods, and the Spearman correlation coefficient was utilized for analysis of the relationship between numerical variables. The results are presented in the tables.

## 3. Results

### 3.1. Characteristics of the subjects

Fifty hospitalized patients with COPD acute exacerbation (AECOPD group composed of 46 male and 4 female) were recruited in this study. Thirty-three of the subjects (31 male and 2 female), were reevaluated after discharge, in the stable period of COPD (SCOPD group). The baseline demographic and clinical characteristics of the patients involved are summarized in Table 1. Seventeen patients could not be evaluated. The withdrawal of these 17 subjects was due to the following factors: i) Acute exacerbation of COPD (11 subjects); ii) death (two subjects); iii) discontinued follow-up (four subjects).

**Table 1:** Characteristics of the subjects. FEV1: forced expiratory volume, FVC: forced vital capacity, CRP C: reactive protein, WBC: white blood cell count.

	Group 1	Group 2	
	(Acute exacerbation of COPD)	(Stable COPD)	
	n= 50	n = 33	P value
Mean age (year)	60.88 ± 7.74	61.06 ± 8.60	0.921
Sex (female/male) (N, %)	4 / 46	2/31	0.738
Body mass index (kg/m2)	24.78 ± 5.44	25.44 ± 5.23	0.590
Current/former smokers	4 / 46	2 / 31	0.738
Smoking index (pack/year)	24.78 ± 5.44	25.43 ± 5.23	0.590
Mean FEV 1 % pred	30.33 ± 12.58	30.92 ± 10.49	0.828
Mean FVC % pred	47.3 ± 16.44	47.9 ± 15.09	0.874
Mean FEV1/FVC	47.35 ± 11.25	47.79 ± 11.58	0.869
Mean pO2 (mmHg)	59.87 ± 15.40	80.68 ± 13.23	<0.001
Median CRP (mg/L)	22.75 ± 51.75	4.9 ± 10. 5	<0.001
Median WBC (/ml)	10.01 ± 3.69	9.27 ± 2.26	0.011

### 3.2. Serum SP-D levels

Serum SP-D at exacerbation (SP-D-AE) was higher than SP-D at stable period (SP-D-S) (86.20 ± 25.75 and 63.69 ± 36.64 ng/mL, respectively; median ± IQR: P < 0.001).

### 3.3. Serum SP-D level and other parameters of the groups

There was a statistically significant difference between the exacerbation and stable periods of COPD in terms of median serum SP-D, CRP, WBC (White Blood Cell), neutrophil, eosinophil, pCO2 (partial carbon dioxide pressure), and pO2 (partial oxygen pressure) values (P < 0.05), which are summarized in Table 2.

**Table 2:** Comparison of serum SP-D median differences between groups. SP-D: surfactant protein D, CRP C: reactive protein, WBC: white blood cell count, PMNL: polymorphonucleer leukoycte, pCO2: partial pressure of carbon dioxide, pO2: partial pressure of oxygen.

	Group 1	Group 2	P value
	(Acute exacerbation of COPD)	(Stable COPD)	
	n = 50	n = 33	
Serum SP-D (ng/mL)	86.20 ± 25.75	63.69 ± 36.64	<0.001
CRP (mg/L)	22.75 ± 51.1	4.9 ± 10.5	<0.001
WBC (/µL)	10.015 ± 3.69	9.275 ± 2.26	0.011
PMNL (/µL)	7.23 ± 3.33	5.655 ± 2.22	0.004
Eosinophil (/µL)	110 ± 170	180 ± 211	0.009
pCO2 (mmHg)	48 ± 15	44 ± 9	0.011
pO2 (mmHg)	54 ± 17	80 ± 16	<0.001

### 3.4. Serum SP-D level and number of admissions and hospitalizations in the past 1 year

There was no statistically significant relationship among the COPD exacerbation period serum SP-D level, the number of admissions in the past 1 year, and the number of hospitalizations in the past 1 year (P > 0.05).

### 3.5. Correlations between SP-D levels and readmissions

22 (44%) of the patients had emergency admission in the first month. There was a statistically significant difference in exacerbation median SP-D value according to first-month admittance status (P < 0.05). Based on this, the median exacerbation period SP-D level of the patients admitted in the first month was statistically significantly higher than that of the patients who were not admitted (P < 0.05).

The rate of emergency admission because of COPD within 1, 2, and 3 months after discharge was 44%, 36%, and 24%, respectively. 27 (54%) of the patients had emergency admission in the 12-month follow-up and SP-D-AE median were 87.1 ng/ml. There was a positive correlation between the rate of emergency admission and serum SP-D-AE levels during the 12-month follow-up period after discharge (P = 0.04 (r = 0.29)).

### 3.6. Correlations between SP-D levels, eosinophil count, eosinophil ratio and emergency admission, rehospitalisation, illness stage, and mortality

Although there was no statistically significant difference according to the first-month hospitalization status with respect to medians CRP, neutrophil, and neutrophil% values (P > 0.05), there was a statistically significant difference with respect to medians exacerbation period SP-D, eosinophil, and eosinophil% values (P < 0.05). While SP-D-AE levels of the hospitalized patients were significantly higher, their median eosinophil and eosinophil% values were statistically significantly lower (P < 0.05) (Table 3).

**Table 3:** Analysis of the difference in terms of parameters according to the 1st month hospitalization status. SP-D: surfactant protein D, CRP C: reactive protein, WBC: white blood cell count.

	1st month hospitalization	Mean	Median	IQR	Min	Max	P value
Exacerbation SP-D (ng/ml)	Yes	109.07	98.24	26.34	77.53	183.77	0.015*
	No	80.23	84.01	26.91	18.84	154.73	
CRP (mg/ml)	Yes	85.82	61.00	144.80	0.90	234.0	0.106
	No	36.34	17.90	42.30	14.64	220.0	
WBC (/µ)	Yes	11.929	12.070	6.850	5.920	23.040	0.232
	No	10.421	9.630	3.120	3.870	25.380	
Neutrophil (/µ)	Yes	9.548	8.300	5.460	2.880	21.960	0.099
	No	7.215	7.000	3.570	1.590	24.030	
Neutrophil %	Yes	75.27	75.00	18.00	48.70	95.0	0.190
	No	68.11	68.60	18.00	8.00	94.0	
Eosinophil (/µ)	Yes	56.36	50	110	0	170	0.026*
	No	172.82	140	220	0	850	
Eosinophil %	Yes	0.62	0.40	14.61	0.00	1.8	0.038*
	No	1.74	1.50	2.60	0.00	6.5	

There was no statistically significant difference among the second and third months and sixth-month readmission status with respect to medians exacerbation period SP-D, CRP, WBC, neutrophil, neutrophil%, eosinophil, and eosinophil% values (P > 0.05).

There was a correlation between the rate of emergency admission and serum SP-D- AE levels during the 12-month period after discharge (P = 0.04 (r = 0.29)). A correlation was detected among the rate of emergency admission and illness stage, FEV1, and mMRC score during the 12-month period after discharge (P = 0.0001 (r = 0.5), P = 0.005 (r = −0.4) and P = 0.01 (r=0,38), respectively).

All patients received systemic corticosteroid therapy during hospital stay. Two patients needed for ICU stay, and no patient needed mechanical ventilation. A total of 19 patients need noninvasive mechanic ventilation (NIMV) during hospital stay. There was no statistically significant difference between NIMV taking patients serum SP-D-AE level and others (P > 0.05). There was no correlation between SP-D and length of hospital stay. (P > 0.05) 

In our study, 10 patients died in follow-up period. There was no difference between the SP-D levels (P = 0.2), although the number of patients is low. Therefore, we can say that SP-D cannot be used to show the mortality of COPD.

## 4- Discussion

Acute exacerbations observed throughout the course of the disease in patients with COPD are a major cause of morbidity and mortality (10). Some studies have shown that an increase in airway inflammation may have a role in the frequency of acute exacerbations (11,12). Thus, the prediction of exacerbations and treatment of patients during the stable period for preventing exacerbations are important. It is expected that there will be an increase in SP-D during COPD exacerbations, which is a specific inflammation marker of the lung. As shown in previous studies, SP-D was elevated during acute exacerbation compared with the stable period in our study. In addition, there were a large number of studies involving thrombocyte, leukocyte, CRP, and fibrinogen levels in exacerbation of COPD as inflammation markers (3,8). The difference of SP-D from these inflammatory markers is that it is specific to the lung. For this reason, we think that SP-D may be superior to the others for predicting exacerbation. Supporting this, we have shown that only CRP levels increase in exacerbation that is correlated with SP-D. Although the number of patients is low, SP-D seems to be superior to CRP in the early period after discharge to show applications and 12-month application status. But in daily practice, CRP measure is cheaper and easier than SP-D.

Patients included in this study were followed up for 12 months after discharge with respect to hospitalization and mortality. There was a positive correlation between the rate of emergency admission and serum SP-D-AE levels during the 12-month follow-up period after discharge. There are different results associated with SP-D levels and exacerbation and hospitalization rates in published studies (12,13). In our study, the results show that high levels of serum SP-D-AE level, which is an indicator of inflammation in COPD exacerbations, indicate higher emergency admission during the 12-month period after discharge. SP-D is an independent predictor of readmissions.

Recent studies indicate a weak correlation among FEV1, symptoms and impairment of a patient’s status. In addition, deteriorating airflow limitation is associated with an increasing prevalence of exacerbation, hospitalization, and risk of death (1). During the long-term follow-up period, we found a significant correlation between the stable period FEV1 values and the rate of emergency admission. 

The relationship between the level of inflammation in COPD exacerbation and mortality rates has been studied before. Lomas et al. found no significant difference between the deceased patients and survivors within the 6-month follow-up period after the COPD exacerbation in terms of serum SP-D levels (12). In a study by Garcia-Aymerich et al., patients with COPD were followed up for 1 year, and there was no correlation between COPD mortality and inflammation marker levels (14). 

As the number of exacerbations and/or the frequency of exacerbations requiring hospitalization increases, the mortality of the disease increases. Exacerbation severity may be independent of COPD severity (10). One of the factors determining the course of the exacerbation is the necessity of mechanical ventilation (noninvasive and/or invasive). Mortality is higher for those need mechanical ventilation support in COPD exacerbation. We did not find any difference in SP-D and length of hospital stay among our patients who received and did not receive NIMV support. NIMV SP-D does not show the need for noninvasive mechanical ventilation and exacerbation severity.

Our study was designed to evaluate the relationship between the SP-D level during the exacerbation and severity of the exacerbation and early mortality. However, the most important limitation of our study is the number of patients. In conclusion, although our patient group is small, SP-D in COPD exacerbation is a significant marker of inflammation of the blood level rising compared to the stable period, and it can be used to predict an emergency admission after hospitalization. However, an evaluation based on the early and late mortality does not appear to be appropriate for the present study.

## Acknowledgments

The authors would like to thank Turkish Respiratory Society for providing professional statistical support.

## References

[ref0] (2018). Initiative for Chronic Obstructive Lung Disease (GOLD).

[ref1] (u2dec). Serum surfactant protein D is steroid sensitive and associated with exacerbations of COPD. Eur Respir J.

[ref2] (2015). Biomarker development for chronic obstructive pulmonary disease from discovery to clinical implementation. Am J Respir Crit Care Med.

[ref3] (2014). Surfactant protein D in chronic obstructive pulmonary disease (COPD). Recent Pat Endocr Metab Immune Drug Discov.

[ref4] (1996). Interactions of recombinant human pulmonary Surfactant Protein D and SP-D multimers with influenza A. Am J Physiol.

[ref5] (1995). Pulmonary surfactant protein D in sera and bronchoalveolar lavage fluids. Am J Respir Crit Care Med.

[ref6] (2007). Circulating surfactant protein D as a potential lung-specific biomarker of health outcomes in COPD: a pilot study. BMC Pulm Med.

[ref7] (2012). Serum surfactant protein D: Biomarker of chronic obstructive pulmonary disease. Dis Markers.

[ref8] (2014). Serum surfactant protein D during acute exacerbations of chronic obstructive pulmonary disease. Dis Markers.

[ref9] (2010). Susceptibility to exacerbation in chronic obstructive pulmonary disease. N Engl J Med.

[ref10] (2015). The role of serum SP-D in COPD. Qatar Med J.

[ref11] (2009). Serum surfactant protein D is steroid sensitive and associated with exacerbations of COPD. Eur Respir J.

[ref12] (2013). Value of serum and induced sputum surfactant protein-D in chronic obstructive pulmonary disease. Multidiscip Respir Med.

